# Exploring External Factors Affecting the Intention-Behavior Gap When Trying to Adopt a Sustainable Diet: A Think Aloud Study

**DOI:** 10.3389/fnut.2021.511412

**Published:** 2021-02-19

**Authors:** Leonie Fink, Carola Strassner, Angelika Ploeger

**Affiliations:** ^1^Department of Food Nutrition Facilities, Münster University of Applied Sciences, Münster, Germany; ^2^Faculty of Organic Agricultural Sciences, Specialized Partnerships in Sustainable Food Systems and Food Sovereignty, University of Kassel, Kassel, Germany

**Keywords:** intention-behavior gap, sustainable diet, think aloud, nutrition behavior, behavioral factors, sustainable food systems

## Abstract

Not least from an ecological and health perspective, it can be posited that a broader part of consumers should practice sustainable diets. People who are already willing to do so are often confronted with the intention-behavior gap, caused by a range of internal and external factors. To eliminate these barriers requires a deeper and more comprehensive understanding of these factors and their interplay. Therefore, a think aloud study with 20 adult German participants was conducted to explore the four chosen external factors of availability, education, advertising and price. Furthermore, questionnaires for all four factors were handed out and a follow-up interview was conducted to gain additional qualitative data. Results show that these four external factors seem to have a major impact on the intention-behavior relation. According to the participants all factors interact in some way with other internal and external factors, making practicing sustainable diets a complex activity. In conclusion, the four external factors availability, education, advertising and price need to be addressed by various stakeholders within our food systems in order to move forward in the process of making sustainable diets practicable and sustainable food systems firmly established.

## Introduction

The understanding that our daily practiced diets have an impact on our environment and influence the world's climate, water quality, soil conditions and biodiversity is not new (1–6), similarly the fact that our diets can take a dual role as cause and prevention of human and environmental health (7). The compilation and composition of a diet is related to what a food system offers. In turn the demand on food, created by diets, has a direct impact on what the food system delivers (8). Therefore, diets can play an important, if not a key role when it comes to eliminating the threats to the environment and achieving the UN's sustainable development goals. Along with our diets, our food systems need to become more sustainable in the face of current and future challenges such as health and environmental issues (9, 10). The close link between a food system and a diet in terms of sustainability becomes also evident by having a look at their definitions:

“*A sustainable food system (SFS) is a food system that ensures food security and nutrition for all in such a way that the economic, social and environmental bases to generate food security and nutrition of future generations are not compromised.”* (11).

“*Sustainable Diets are those diets with low environmental impacts which contribute to food and nutrition security and to healthy life for present and future generations. Sustainable diets are protective and respectful of biodiversity and ecosystems, culturally acceptable, accessible, economically fair and affordable; nutritionally adequate, safe and healthy; while optimizing natural and human resources.”* (12).

**Figure 1:**
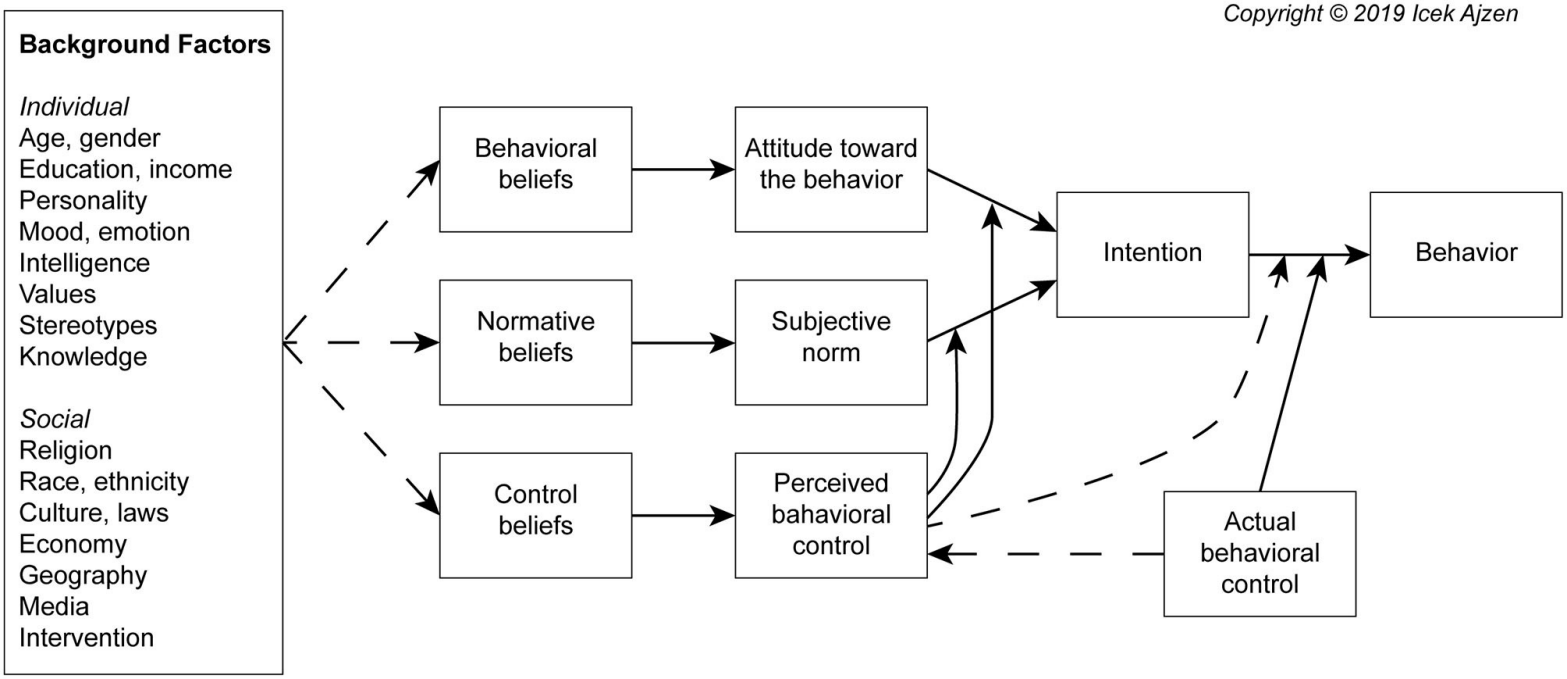
Theory of Planned Behavior (TPB).

We can see that both definitions address, amongst others, the dimensions health, environment, economy and society and that they are interdependent (13). Especially their extent of sustainability depends on each other (14). This interdependence can be illustrated by the current state of research on the Mediterranean diet as one example of a sustainable diets (15). The framework for revitalizing the Mediterranean diet provides four sustainable benefits that are strongly interdependent and resulting from the diverse Mediterranean food systems: (1) well-documented nutrition and health advantages, preventing chronic and degenerative diseases and reducing public health costs; (2) low environmental impacts and richness in biodiversity, reducing pressure on natural resources and climate change; (3) positive local economic returns, reducing rural poverty and (4) high social and cultural food values, increasing appreciation, mutual respect and social inclusion (16, 17). Of course there is not one Mediterranean diet, because it varies from country to country while considering different local environments and economies, as well as social and cultural features (18, 19). Therefore, it can be said that the Mediterranean diet is a complex web of nutritional, cultural, historical, economic, political and religious aspects that all somehow interact within Mediterranean food systems when the diet is being practiced (20). Despite the overall complexity that comes with today's understanding of sustainable diets we need to make dietary decisions that preferably don't jeopardize our health, environment, economy and society (21). In order to proceed a corresponding sustainable development, behavior changes will be needed (22), as well as strategies that promote sustainable diets in different contexts worldwide (23). A starting point for awareness raising is to include sustainability in food based dietary guidelines (21, 24) as well as to promote that diets with a higher amount of plant-based food and a lower intake of meat and dairy food products produce less greenhouse gas emissions (25, 26) and bring a range of other health, environmental, economic and social benefits (1, 27). The importance of practicing sustainable diets is now increasingly widely acknowledged (28, 29) and partly consumer behavior is already changing toward purchase of sustainable food products because of raised awareness (30). However, most of the time we are struggling with nourishing ourselves sustainably, even if we consciously formed the intention to do so. In general, the phenomenon of not acting as we intended is called the intention-behavior gap. In Figure 1 we can see that according to Ajzen's Theory of Planned Behavior (TPB) our formed behavioral intention is based on the attitude toward the behavior, subjective norms and perceived behavioral control. The formed intention is then not always directly translated into behavior (31, 32). Ajzen states that “People can be expected to act on their intentions only to the extent that they have sufficient control over the behavior in question.” (33). Whether people's intention for a specific behavior will successfully be performed is therefore dependent on confidence and commitment toward the intention and the perceived and actual behavioral control. There are internal and external factors which act as barriers by interfering with our behavioral control. Internal factors such as the general ability to exercise behavioral control, information, skills, abilities, will power, emotions, stress and compulsions can influence our behavioral control as well as external factors such as time, opportunity and dependence on others (31). People with a higher perceived control of influencing factors are likely to translate their intention into the foreseen behavior (34). Barriers to or influencing factors on practicing a sustainable diet can be for example availability of food, lack of information, poor presentation or food prices (35).

If it is already difficult for people who have formed a positive intention to act decisively, what about those who still need to be convinced? To tackle unsustainable food consumption, we need to promote sustainable diet practices on an individual level. On the one hand this includes increasing the awareness of sustainable diets and on the other hand exploring determining factors for practicing sustainable diets (36). Food system improvements, for example implementing sugar or fat taxes, and innovations are necessary to promote and enable people's behavior change (37). People need support to close the intention-behavior gap and to be able to continuously practice sustainable diets. To get to this point we need a better understanding of the factors influencing our nutrition behavior. Therefore, this paper seeks to explore four chosen external factors that are involved in causing the intention-behavior gap when it comes to practicing sustainable diets, and to get a broader understanding of their interrelationships. Then we can better make valuable recommendations for policy, stakeholder and consumer action within food systems, for closing the intention-behavior gap. We focus on the external factors because addressing them within our food systems can be a good starting point to create the best conditions enabling people to nourish themselves sustainably. On this account a mixed method approach is conducted to gather qualitative and quantitative data. The analysis allows us deeper insights which in turn enable us to better understand these particular factors and the resulting problem of the intention-behavior gap and how to handle it in future.

## The Think Aloud Approach

To learn more about the external influencing factors and their effects, it can be helpful to know what people are thinking. One method that helps researchers to do so is the think aloud approach which can offer us insights into people's thoughts, feelings and intentions (38). According to scholarly literature the think aloud method offers the opportunity to gain data that cannot be gained in any other way by any other method. Moreover, qualitative researchers assume that based on think aloud data, models of cognitive processes can be developed (38).

In its original form, the method provides that humans are asked to verbalize their thoughts coming during an activity (task) which is being studied. These verbal utterances then represent the gained qualitative data. However, feelings and daydreams are, according to Ericsson and Simon (39), not considered as hard verbal data, because their clear interpretation and analysis is difficult. The only data that should be evaluated are those about what the subject is doing during solving the task and in what order (40). However, researchers are not always in agreement with that and collect the data that is valuable for their studies (41).

There are four core principles that should be pursued when applying the think aloud approach according to Ericsson and Simon: give subjects detailed instructions for thinking aloud, remind subjects to think aloud and do not intervene otherwise collect and analyze only hard verbal data (40). However, not all research applications of the method follow these core principles (41). It is important for the think aloud process that each subject has an own session in a comfortable and quiet setting and should constantly talk (42, 43). After collecting data through audio or video recording, these verbal protocols need to be transcribed, analyzed and evaluated (42). Ericsson and Simon (39) differentiate between concurrent verbalization during performing a task and retrospective verbalization after performing a task. Retrospective verbalization is further differentiated between immediate retrospection and delayed retrospection (38). In addition, think aloud examinations can be supplemented by triangulation to ensure as complete information gathering as possible (44). Therefore, researchers need a follow-up strategy. This can be, for example, a follow-up interview or a questionnaire (45). As there are different variants of the method and researchers are collecting different kinds of data, the validity of the method stands to question. Although there is little research on the validity of the methodology (46), knowledge about potential threats to validity for concurrent and retrospective verbalizations exists (47). To ensure the validity researchers can remind participants to constantly think aloud and use a control group (47) as well as avoid collecting data via retrospective protocols (48). Aiming for completeness of the verbal protocols, think aloud studies should include other methods (49), as mentioned before.

For a majority of people thinking aloud is new and requires practice, which should be considered when planning an investigation (40, 43).

The think aloud approach finds current application in the fields of problem-solving, language acquisition and reading research, teaching research, decision research, media research and usability tests (38). In addition the method is widely used in marketing and consumer research, advertising effectiveness and purchase decision-making (50). In the food and nutrition sector the method is used to examine behavior at the point of sale (51–53), to explore or identify factors that influence purchasing behavior or food choices (54–59), to improve a questionnaire design (60–62) or to examine nutrition education and interventions (63–66). Researchers are also using the think aloud data to develop or explore hypotheses (49).

## Materials and Methods

This research study was publicized via e-mail distribution lists of students from the Department of Food - Nutrition - Facilities at Münster University of Applied Sciences and oral dissemination in the professional environment of the lead author. Participants, were recruited in January 2019. We selected our participants primarily based on their interest to participate. In accordance with method practice we rather aimed for a medium sample size and proceeded with 20 participants. As with many other research method applications, there is also a heated debate on how many subjects are needed for a think aloud study. Despite that there are studies including about 20 participants or even up to 70 participants and more (42, 45, 54, 55, 57, 64, 66–72), think aloud studies are known for actually not requiring a large sample (70, 73) and may have designs with under 10 participants. This is especially applicable when qualitative topics are explored and no statistical generalizations are made (74). As an incentive we offered each participant five Euros and a sustainable food item (seasonal vegetable from a local organic farm). A written informed consent was received from all participants. In addition, the participants have received a data privacy policy which gives them the opportunity to ask for their data to be deleted at any time.

All interested potential participants were informed by e-mail about what to expect when participating in the study. The think aloud method was already explained in advance, so that people who felt very uncomfortable with the idea had the opportunity to opt out of participation. Along with this information participants received a handout on sustainable diets, which essentially contained the FAO definition (12) and the dimensions and principles of a sustainable diet according to von Koerber (75). By reading the handout, we wanted to ensure that all participants have the same basic understanding of a sustainable diet, even if they may not practice a sustainable diet.

The study took place from January 21 to February 1, 2019. All individual sessions took place in a specially prepared meeting room at Münster University of Applied Sciences. The room offered a pleasant atmosphere (windows, centered work desk) to let the participants feel as comfortable as possible within this setting.

Other than Ericsson and Simon (31) we also wanted to collect data that is not considered as hard data. This is mainly because we don't attempt to build cognitive models, but rather seek to gain insights and also capture feelings and preferences toward the issue under study.

In developing our research design, we decided to proceed with a simplified think aloud approach. Since our research focus lies on the behavioral control influencing external factors and not on the pure action process itself, it was considered suitable for us to use video simulations of typical daily situations. This approach is often used in client simulation think aloud studies (42). We produced four videos in each of which one external factor is responsible for creating the intention-behavior gap. Based on previous research (76, 77) we decided to choose the external factors availability, education, advertising and price. Availability, education and advertising were three of the most mentioned factors responsible for the intention-behavior gap. In addition, we added the factor price, as this is often an obstacle for people to consume sustainably produced food like organic food (78). Of course, there are several other external factors such as time, food packaging and social environment that need to be examined in more detail. With regard to the first application of the method combination, we initially decided on the four chosen factors. The videos were produced in the style of cut-out animation. This is a film technique in which cardboard cut-outs (figures and other items) are moved by hand on a surface and put into relation to each other. This procedure is filmed in live-action, cut, set to music and a narrator's voice is added to tell a story, or explain something. The task of the participants was therefore to watch the videos one after another and simultaneously verbalize all their thoughts. Our think aloud guideline for the participants included the following procedure:

- Welcome participant and offer a glass of water
- Express thanks for willingness to participate
- Hand out a questionnaire to collect demographic data
- Give a brief instruction to the procedure and explain each step
- Provide time for any queries
- Start warm-up practice
- Provide time for any queries
- Start the think aloud session (including supplementary questionnaires for each external factor/video). This includes the following text: “Please remember to constantly verbalize all thoughts that come to your mind.”
- Follow-up interview; including four questions
- Reflect the study procedure with the participant
- Hand out a feedback questionnaire concerning the method application
- Express thanks for participation and present the incentive
- Say goodbye to the participant.

We stayed absent from the room during the actual think aloud procedures and did not intervene at any time. We also supplemented our think aloud data with a questionnaire and a follow-up interview. Mainly, we did so to ensure that we get data if the participants wouldn't think aloud sufficiently for analysis purposes (back-up plan) as well as getting specific information on subtopics. We used the questionnaires to get specific information for each of the four factors. For this purpose, we focused on the possible influence of the factors on the intentions-behavior gap and on possible ideas for avoiding the emergence of an intention-behavior gap based on the factors. The latter ties in with the work of our first study (77). The interview was then used to collect general data on the intention-behavior gap, as well as external and internal factors. The questions were partly based on the results and the discussion of the previous work (77). The full sessions were audiotaped and transcribed fully verbatim with the support of the software MAXQDA 2018. With this software we also evaluated the data of the think aloud protocols, the qualitative part of the questionnaires and the follow-up interviews. We decided to first proceed with an inductive coding of the material to structure the material and then we conducted a qualitative content analysis.

## Results

### Study Sample

The final study sample included a total of 20 participants comprising thirteen women (65%) and seven men (35%). Additional study sample characteristics are shown in Table 1. Not all participants were students from Münster University of Applied Sciences, only 14 participants were enrolled. In response to the question of whether the participants try to practice a sustainable diet, 17 responded with yes and 3 with no.

**Table 1:** Socio-demographic profile of the sample.

**Socio-demographic characteristics**	**Total number (*n* = 20)**	**Percentage (%)**
**Sex**		
Female	13	65
Male	7	35
**Age**		
18–19	1	5
20–29	10	50
30–39	8	40
40–49	1	5
**Marital status**		
Single	18	90
Married	2	10
Separated/ Divorced	0	0
Widowed	0	0
**Household composition**		
1 person	7	35
2 people	8	40
3 people	1	5
4 people	3	15
8 people	1	5
**Education**		
Abitur (high school diploma)	9	45
Bachelor degree	5	25
Master degree	5	25
Diploma degree	1	5
**Monthly income (net)**		
<500 €	3	15
501–1,000 €	5	25
1,001–2,000 €	6	30
2,001–3,000 €	2	10
3,001–4,000 €	2	10
>4,000 €	1	5
Prefer not to say	1	5

### Results From the Think Aloud Method Application

Each participant provided a think aloud protocol for every chosen external factor (availability, education, advertising and price). Some are longer or more detailed than others. Overall, however, it can be said that almost no participant had difficulty expressing their thoughts aloud. Three participants were conspicuous in that they verbalized only a few words or short sentences. The authors have translated the quotes from German into English, keeping to the original even where sentences are incomplete or the quotes are grammatically incorrect.

Having a look at the inductive coding of the think aloud protocols brings us insights to each of the external factors. In Table 2 we can see that within the protocols for availability there are factors that come up very often like availability (50 codings), planning (22 codings), hunger (21 codings), information (14 codings), personal norms and values (14 codings) and time (13 codings). Based on the codings and content analysis we can state that a higher availability of sustainable food is needed, so that people have access to buy it. Especially when people are hungry and on the go, and are short of time, it would be helpful, if there is a high and fast availability, otherwise other offers such as widespread fast food chains will (or must) be used instead.

P4: “(…) And then you feel bad, but there is just no other way and yes, because nothing is available. If there is nothing offered, then I cannot buy something. And if I haven't planned that, then that is very unfavorable and then sometimes there is no other way and yes, then you have to face the intention-behavior gap. You have to eat something anyway, because you're hungry.”

**Table 2:** Inductive coding of the think aloud protocol data.

**Main code**	**Sub code**	**Availability coding**	**Education coding**	**Advertising coding**	**Price coding**
External	Advertising	0	0	38	2
External	Availability	50	8	14	12
External	Education	0	9	2	1
External	Information	14	16	3	1
External	Labeling	0	13	2	0
External	Packaging	0	2	0	0
External	Politics	2	4	1	5
External	Price	4	13	1	37
External	Social environment	0	3	2	15
External	Time	13	1	4	0
External	Transparency	2	9	3	0
Internal	Consciousness	5	13	14	12
Internal	Finance	1	3	0	32
Internal	Health	5	2	7	3
Internal	Hunger	21	1	18	0
Internal	Knowledge	7	28	2	5
Internal	Personal norms and values	14	20	8	18
Internal	Planning	22	0	9	9
Internal	Taste	3	0	7	1
Internal	Willpower	7	1	15	7

One important factor seems to be the planning of the daily diet and to gather information in advance where to find something suitable to eat when one is out of home.

P13: “(…) And you should at least take care of your food-supply half or three-quarters of a day. Then look, um, where you can dine. In addition, she [character in video] could have informed herself in advance. Or just ask a stranger/ local person.”

As already mentioned in the above quotes, the provision of information is also of decisive importance. Respondents indicated that people need access to information about where to find sustainable food offers in a supermarket or restaurant.

P19: “(…) What is difficult is that many facilities such as supermarkets and restaurants have a rather conventional offering and not focus on organic and sustainability. Information from locations may also be poor, poorly given. Maybe it would be helpful if you would somehow use apps or something similar. Available are conventional foods. That, um, what has a lot of money and is directly available and is on your way. And that is the economic system which is just established, it's not yet operating that the focus is on organic or local. And in the meantime, the pressure of time that arises in our society [meaning having lack of time], doesn't contribute to facilitate that one could somehow inform himself or herself in detail where to go [to buy sustainable food]. (…).”

For education there are coded factors like education (nine codings), knowledge (28 codings), personal norms and values (20 codings), information (16 codings), labeling (13 codings), price (13 codings) and consciousness (13 codings) that come up quite often within the protocols. It is striking here that education itself doesn't have the highest number of the codings, as it is the case with the other three external factors. Knowledge, information, labeling, personal norms and values and consciousness have more codings, as they may be included in the understanding of education from the participants' point of view. At best, education leads to knowledge. This is what a number of quotes indicate, and, furthermore, that people need to deal with education offers themselves to gain knowledge.

P3: “(…) Of course you have to know that, or make the transfer that it [vegetables] usually only grows in the summer. Exactly, and not in the greenhouse. That this is, of course, not good for the life cycle assessment, you have to know that too. That means you have to deal with it. (…)”

According to the participants, knowledge can then also be key to buy the “right” food items and know how to prepare them.

P10: “(…) So, education is very important. I notice that with my roommates. They have no idea and buy, um, tomatoes from Spain in winter. And, um, yes/ I wouldn't do that, or I fundamentally changed my behavior and only buy local food. (…) I buy in season, but also because I simply have the knowledge of what vegetables grow when and which fruits can be eaten in the winter, um, anyway, because they can be stored, as for example apples. Many students don't even know how to prepare seasonal or local vegetables. They don't know that at all. For example, they don't know what to do with a rutabaga, or they don't know how to prepare fennel and therefore they don't buy it.”

Within educational work, information about food labeling needs to be incorporated according to the participants, and in general comprehensive food labeling needs to be established. Moreover, there must be more education about nutrition and food systems in schools, so that people are already trained and know how to act when they are adults, as exemplified by the following response.

P11: “(…) So that's a classic example for the need for comprehensive labeling and, um, appropriate labeling. And that must be much more taught, um, also in schools and also on the, yes, also on the learning path. Especially in all schools. Um, that should be part of the basic education besides math, German, et cetera. Um, that you know how you can find your way around in the supermarket. I think these two ways should merge. And, um, on the one hand people should be enlightened about such things already in school. And, um, on the other hand, that this label jungle is also somehow reduced, but with good standards.”

Furthermore, participants suggest that even supermarkets can support education by providing their customers with information about food labeling and the product itself. In this way there is a direct confrontation with the product's background at the point of sale.

P19: “(…) The economic system states that cheap food is better, because it doesn't cost that much. It would be good, if there would also be information about the product itself and what the labeling means. An alternative would be maybe that supermarkets would provide information by somehow putting up posters or stand up displays, where information is generated. Then people can have access during shopping. (…) And I think that somehow you have to confront people directly and that they cannot generate information by themselves. (…)”

The most common codings within the think aloud protocols for the factor advertising show the following distribution: advertising (38 codings), hunger (18 codings), willpower (15 codings), availability (14 codings), consciousness (14 codings), planning (9 codings) and personal norms and values (8 codings). Participants said that advertising is subconsciously influencing people's behavior and it evokes needs that are otherwise not present or relevant.

P11: “So, advertising should be significantly more regulated. So that unhealthy foods should not be promoted that way, because kids can see it [advertisement] too. There is also a significant imbalance between food products that are promoted by advertising, between fresh and healthy foods and, um, yes, snack food. Through this imbalance also needs are arising that otherwise wouldn't exist. Yes. Yes, that's definitely a big influence, if not a very big one [influence]. Um, I think in many ways also underestimated [advertising]. Because that subconsciously just triggers so much over the years. You grow up with it. And I think you would have completely different eating habits, if you were not confronted with so much advertising from your childhood onwards. (…)”

Also, participants indicate that it seems to be very difficult to resist advertising because it has such a strong power and influence on people, especially when advertised foods are available everywhere.

P14: “(…) Um yes, but it is already proven that, um, no matter how much we are aware of what advertising triggers in us, it still has an effect because it affects a lot our subconscious, exactly, it effects a lot in the subconscious. (…) So, as I said, it still has an impact on us when we, um, when we are confronted with advertising and then, in that moment we think, wow I'm hungry, there is such an awesome chocolate bar, I take it. And to control oneself in that situation needs a lot of strength. That's difficult, I think. Yes, well, there are certainly more assertive people than me, but, um, so the majority of society is probably pretty weak in that case. Especially because we are constantly confronted with advertising. Yes. (…).”

Another insight from the data is that according to the participants advertising should be used to promote sustainable diets or sustainable food. This goes together with education or providing people with information; also, that people are able to assess advertising and don't let it have a negative influence on their behavior, as exemplified by the following excerpt.

P19: “Of course, you could also use repeating advertisements in a positive way, to promote sustainable diets. But I believe that the one, who has the most money, is the one who advertises. And these are not really the ones who act ecologically sustainable, um. (…) I think this also interacts with education, um, if you fall for these advertising strategies or not. Or, if you can better assess them or estimate their credibility. Because without this basic knowledge, I think that it will not work.”

Price was the fourth external factor for exploration in our study. The most frequent codings within the protocols are price (37 codings), finance (32 codings), personal norms and values (18 codings), social environment (15 codings), availability (12 codings) and consciousness (12 codings). Participants stated that if people don't have any money, they cannot spend it on a sustainable diet, because it is just not available.

P4: “(…) That situation, if you want to do it, but you haven't enough money for it and then despite that just try to, what is personally important to you/ For example, animal based products, that's always very important to me, that I buy at least these in organic quality. Yes, but if you just don't have any money, then it's just not possible. That's just how it works here in Germany and in other countries I think even more. That's just one big issue, so money, so finances are/ This is really one of the biggest barriers. Because it's [the money] just not there then. You just don't have it then. And then you can turn yourself upside down, but then you just don't have it. So, if, maybe then you have to plan your budget in advance and look how you can divide it. But in that moment, you don't have it, you don't have it and then it is, yes a dealbreaker.”

Participants highlighted that the availability of money also depends on the social environment in which one lives. For example, if people need to provide a family with food.

P11: “Yes, with children, of course, you always have a budget, a certain budget available, just like every household. Exactly. Especially toward the end of the month. That means that the price, of course, plays a role. And if I think of our society, there exists also a great inequality and unfortunately people from lower social strata cannot afford it [to buy higher priced sustainable food]. (…) I believe, as I said, that this is a real problem for families and people from, um, socially difficult strata and from, um, with little income, really little income. (…) So, it depends on the population group. I would say that there is a big difference. For some people it has a very large impact, and for others a very small influence, I would say.”

In the context of food prices, it also seems important to plan in advance and whether a person should reconsider their diet. Then there may be potential savings at some points. For example, if people would rethink their buying behavior and their diet, then their budget can be spent on other food items with a better quality.

P6: “(…) But you can also manage, for example, that you just then don't buy meat and then for this money what you would have paid for the meat you can then just, um, buy other food [sustainable food]. Theoretically you could do it this way. Yes, the price has a very big influence, especially for us Germans. Very, very, I think that the price is the biggest influence on our behavior.”

What is interesting is that people seem to want to save money as a general orientation and that the thought of saving money may be internalized.

P10: “(…) Yes, sometimes I am also happy when I see cheap things and then I always must remind myself to, um, not to see that as a priority, but to buy the food with a higher quality. But sometimes the cheaper products are also finding their way into my shopping cart. Especially if I'm shopping for a group, so if, for example, I have friends come over and I have to cook large quantities, then, um, I often buy cheaper food and no-name products and if I only buy for myself, then the amount is less and then I can also often afford sustainable products. But actually, it's not about if I can afford something, it's more a mental thing. You always want to save money and that's always a bit inside of me. So always this thought of saving money. Um, I think, if I really wanted it, I could afford to buy it. Of course, it would be more expensive and then I would have to lower my sights at other matters of expense. (…).”

One participant stated that for him quality has always the highest priority. When people have this personal norm that the food quality comes first, then the factor of a higher price can be beaten. One further interesting point of view regarding the allegedly higher food prices is that less food waste can be produced in the household due to the lower quantity contents of packaged food.

P20: “(…) Um, so there, um, so in terms of price, for me I always find that it is very difficult. Well, personally, I don't mind that [higher food prices], I always think the quality has to be right and, um, I think Germany is one of the countries, especially compared to other European countries, where the food is really cheap. And even organic food is very cheap in discount supermarkets. Um, for me, as always, the quality must always fit. And that's why the price is not decisive for me. (…) And if I buy organic products, they usually have the same price as conventional products, but there is less in it. Which of course is first not so good, but I think, um, currently, I prefer that because then I throw nothing away. And, um, yes, that is why I often choose the organic product anyway. (…).”

**Figure 2:**
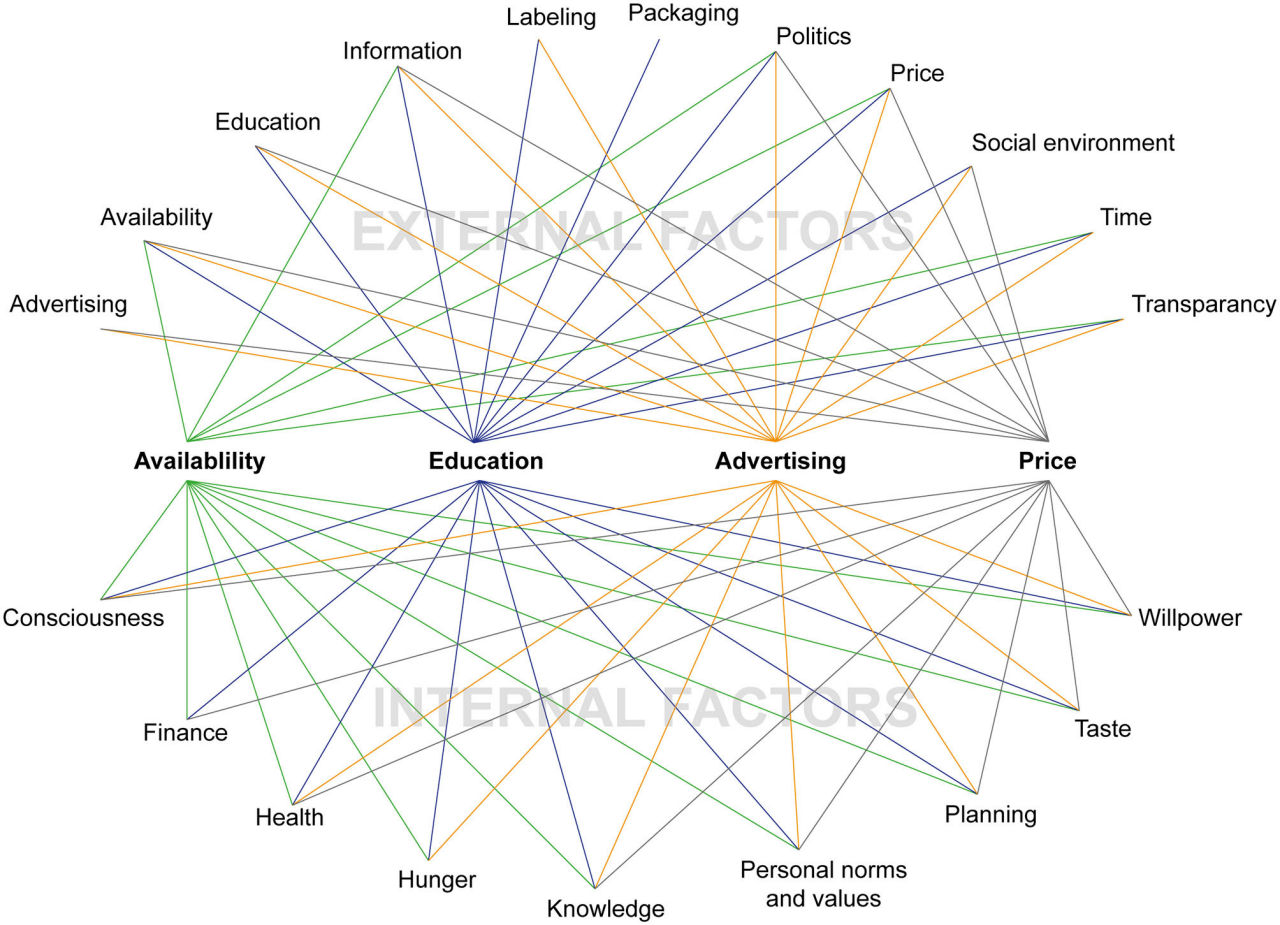
Linkage of external factors to external and internal factors.

Visualizing the results of the think aloud protocols shows, that the four external factors availability, education, advertising and price interact with other external and internal factors (Figure 2).

### Results From the Supplementary Questionnaire

The supplementary questionnaire was filled out for each external factor. It included three questions, two closed questions with multiple choice answers and one open question. With this questionnaire we wanted to gain information about the strength of the influence of each factor on the intention-behavior gap, if the factors are actual barriers for the participants personally and in general and with the last question we wanted to gain ideas how to tackle these external factors, so that they cannot interfere with an intention to follow sustainable diets.

Table 3 provides an overview of the results from the first question. We can see that all four factors were attributed a rather strong influence on the intention-behavior gap. Especially the factors price and education have a strong influence according to the participants' assessment. From all factors considered, advertising seems to be the one that does not have the same strong influence as the other external factors.

**Table 3:** Influence of external factors on causing the intention-behavior gap (*n* = 20).

Q1: In the video the external factor availability/ education/ advertising/ price was displayed. How do you assess the influence of this factor on the intention-behavior gap regarding the practice of sustainable diet?				
	**Availability** ***n*** **(%)**	**Education** ***n*** **(%)**	**Advertising** ***n*** **(%)**	**Price** ***n*** **(%)**
Strong influence	7 (35)	9 (45)	2 (10)	10 (50)
Moderately strong influence	9 (45)	6 (30)	7 (35)	5 (25)
Fairly strong influence	2 (10)	5 (25)	9 (45)	5 (25)
Undecided	1 (5)	0 (0)	1 (5)	0 (0)
Rather minor influence	1 (5)	0 (0)	0 (0)	0 (0)
Moderately minor influence	0 (0)	0 (0)	1 (5)	0 (0)
Minor influence	0 (0)	0 (0)	0 (0)	0 (0)
No influence	0 (0)	0 (0)	0 (0)	0 (0)

For the second question we can see in Table 4 that there is a big difference between the assessment for the participants personally and in general except for the factor availability. Here we can see that it applies as a causing factor in almost all cases personally (95%) and in general (100%). The factors education (100%), advertising (95%) and price (100%) apply almost always in general. Education (45%) and advertising (40%) apply to less than half of the participants personally. The factor price (60%) applies to more than half of the participants personally.

**Table 4:** External factors' contribution in causing the intention-behavior gap (*n* = 20).

Q2: Please indicate below whether this external factor may be the reason for the emergence of a intention-behavior gap for you, personal and, according to your opinion, for the general public.				
	**Availability** ***n*** **(%)**	**Education** ***n*** **(%)**	**Advertising** ***n*** **(%)**	**Price** ***n*** **(%)**
**For you personally**				
Applies	19 (95)	9 (45)	8 (40)	12 (60)
Doesn't apply	1 (5)	11 (55)	12 (60)	8 (40)
**In general**				
Applies	20 (100)	20 (100)	19 (95)	20 (100)
Doesn't apply	0 (0)	0 (0)	1 (5)	0 (0)

Quotes for the third question can be found in Table 5. Ideas for the factor availability target a higher availability of sustainable food and a better individual planning of the daily diet to avoid a lack of availability. For example, precooking and taking food from home with. Along with planning goes information gathering via tools such as mobile applications that can show persons where to find sustainable food offers.

**Table 5:** Participants' ideas for preventing the intention-behavior gap.

Q3: Can you spontaneously give any tips on how to prevent the emergence of an intention-behavior gap caused by the corresponding factor and if yes, how?	
	**Idea quotes (translation provided by authors)**
**Availability**	Offer more “healthy fast food” in cities, train stations, highways
	City apps offer targeted information for sustainable, healthy and readily available nutrition options
	Better planning of everyday nutrition - do not leave it to chance
	Meal prepping and trying to have sustainable snacks always with you, especially if you know that you are out of home
**Education**	The aspects of nutrition, health and sustainability should already be treated in elementary schools regularly
	Educational work in the media
	Political local implementation strategies that directly affect the population
	Public information, e.g., on the products or on stand-up displays in supermarkets
**Advertising**	Be aware of the impact advertising can have. Counter it.
	Promote healthy and sustainable products more strongly; So that people are not only bombarded with advertising for unhealthy food products but also with desirable products
	Consumers can use tricks to protect themselves from advertising, such as not shopping hungry and carrying a grocery list
	One can also use this positively and advertise sustainable products and place them cleverly for shopping
**Price**	Plan and manage your monthly budget at the beginning of the month
	Sustainability communication: why are the products more expensive and what
	Internalization of costs of conventional foods; This makes them more expensive and organic food able to compete
	Educational work on consuming in general. People consume so many unnecessary things that it follows that the money cannot be spent on food

To prevent the intention-behavior gap caused by the factor education, participants see more educational work especially in schools and through media as promising, as well as political support for implementing this, but also providing information about the food products directly at the point of sale.

Looking at ideas for tackling advertising as an influential factor, participants stated that people must be aware of advertising and the effects that come along with it and try to avoid getting confronted with food advertisement. Ideas were also raised to use advertising for sustainable food items and to promote them in the same way as is done currently with sweets and other food items.

Regarding the price, participants voiced ideas to strictly plan the monthly budget for food if it is rather tight and to use educational work so that people know and are aware of why sustainably produced food is more expensive (and are then willing to pay the higher prices). Additionally, they suggested regulating food prices by political action, so that they are reflecting the real price. Especially when applied to non-organic products.

### Results From the Follow-Up Interview

The follow-up interview directly after the think aloud session included four questions to gain more information about external factors and their influence on practicing a sustainable diet and comparing their influence on the intention-behavior gap to the influence of internal factors.

The first question (Q1: Would it be easier to consistently implement a sustainable diet or more likely that the intention-behavior gap doesn't occur, if the external factors presented (availability, education, advertising, price) wouldn't create any barriers?) was answered by all participants in the affirmative. Most of them said that these external factors are the most decisive ones because they dictate the framework conditions.

P5: “Well, yes that would be easier. I think these are the basic reasons why it does not work. For now, I cannot think of other reasons why it does not work. (…)”

One participant added that probably nobody intentionally doesn't behave sustainably.

P7: “Yes, in my opinion it would be easier, yes definitely. So, especially if people were educated and the price is just right. I think everybody wants to do something good or wants to act sustainably and if this problem is not so big, depending on the persons how big the problem is/ But, um, I think everyone wants to try to live as sustainably as possible, so I think no one wants to buy no organic food on purpose.”

Other participants focused on factors such as the price and availability of food.

P8: “Yes, absolutely. So, for me personally in any case, because for example, I currently earn no money and then it is partly really difficult due to the price, if you don't have so much money available and organic is sometimes much more expensive. (…).”

With the second question (Q2: What other potential external factors are you spontaneously aware of, that can create an intention-behavior gap regarding the practice of a sustainable diet?) we asked for possible other external factors that can have an influence on our behavioral control and thereby contribute to the intention-behavior gap. Twelve people mentioned the social environment.

P18: “So externally means, so for example, if I have friends or acquaintances in the clique, who are also totally in to it and say, uh no way, you have to buy the fair trade bananas and not the super cheap ones. That is clearly an important external factor for me. Such examples, or if there are role models, like for example parents for their children. (…).”

Three people stated that the food packaging can also have an influence. It depends mainly on whether the packaging is useful and necessary, especially for vegetables and fruit.

P8: “(…) Above all, um, what I find difficult is to say yes, organic is great, but for example, organic is partially wrapped in plastic and the other stuff that isn't organic isn't [wrapped in plastic], and then you get in to a conflict. Yes, you actually want to buy it, but at the same time it's packed in plastic, which makes no sense at all, yes.”

Two people have come to think that the factor time can also have an impact on our intention-behavior relation.

P9: “(…) So that and the factor time, of course. If you are in a hurry. So, you don't want to prepare anything or you are just about to leave, uh, then you sometimes take pre-packaged and convenience food. (…).”

For one participant the attractivity, here meaning the appearance of the food itself, the shopping atmosphere and the product presentation can have an influence on buying behavior.

P2: “Um, the appearance of the food. That's silly, but in most organic markets, everything is so beautifully presented and, um, polished to a high gloss. And if you sometimes see such a crooked organic carrot, you might think “Huh?”. Um, the ambience (…) creates a feel-good shopping atmosphere. (…) It's just a presentation of the products that you are willing to pay the higher price because you think it's worth it. Yes, product presentation. (…).”

The external factors mentioned might be supplemented with the factor labeling (named by one participant), in which transparency plays a role, according to the following statement.

P8: “(…) But that one cannot tell from the product's appearance, um, whether it is sustainable or not. Although there are labels, but there are many (…) and sometimes very misleading [talking about the food labels]. And um, that's why it's sometimes difficult. (…).”

The third interview question (Q3: From previous research we have gained the impression that external factors are often made responsible for failure to implement sustainable diets. To what extent do you share the attitude of the following the motto: The others have to change something first before I can change something?) wasn't answered clearly all the time. Seventeen times participants said that they don't agree with that statement.

P17: “I do not share this, because I want to change that [talking about diet] for myself and maybe for my children and grandchildren. A grandchildren-suitable world and so on. (…).”

At the same time we have also five participants that agree with the statement, that other things have to change first before they can change something.

P12: “Um, actually I share that, because I personally think that, um, the consumer is imposed too much responsibility. (…) The individual is the architect of their own fortune. And then the individual is also the architect of their own fortune in relation to the food system in which they live and, um/ (…) because, um, I really see the politics as the primary responsibility. (…).”

In addition, two participants have said that in order to answer this question you have to look at it on an individual level.

P1: “(…) Yes, that can be seen individually, I think. I mean it depends/ You cannot generalize that [talking about whether agreeing to the statement or not]. (…) Yes, I would say it is customizable and/ Yes.”

The answers for the last question (Q4: In your opinion, what factors are decisive in causing the intention-behavior gap, external or internal factors (such as willpower and skills)?) show that the opinions about what factors are more decisive, internal or external, are divided. Six participants said that the internal factors are more decisive.

P18: “So, I think the internals [talking about internal factors] are mainly important, because I have to want it first. And if I want it, then I can manage the rest somehow. I would say. That's how I see it. Yes.”

Whereas, four participants said that external factors are more crucial than internal ones.

P11: “(…) Well, I generally believe the external factors actually. Because, um, I believe that if you would ask every human being the question if they want to nourish themselves sustainably, everybody would say yes. (…).”

It is also interesting that half of the participants said that both internal and external factors are equally crucial when it comes to influencing our behavioral control.

P16: “I think both are important. Probably the one then also plays into the other. Because, yes, if everything is expensive or everything is somewhere in another corner of the city, then I also lack the motivation to change something. Well, I do not think you can say that one thing is more important than the other. Both are very important.”

## Discussion

With this investigation we wanted to explore external factors that have influence on people's behavioral control when it comes to practicing sustainable diets. These factors are known to possibly act as barriers and create the so-called intention-behavior gap. Our applied research design offered us valuable data which enabled us to explore each of the factors and gain further understanding of possible interactions and connections as well as how they can possibly be tackled to overcome the emerging barriers.

### The Examined External Factors

#### Availability

First, we discuss our data for the factor availability. From the think aloud protocols it becomes clear that according to the participants it is very difficult to perform a sustainable diet when there is a low availability of sustainable food or no availability at all. This result can be confirmed by the results from the supplementary questionnaire. Participants assessed that availability has a strong influence on causing the intention-behavior gap. Besides, participants (except one) indicated that availability applies to them personally and in general. When people eat out of home, a lack of availability becomes a problem, too, especially when other factors such as strong hunger and lack of time are implicated. At such occasions people usually act fast and not rationally and they are rather buying food that is available fast – even if it's not suitable for a sustainable diet. By analyzing the content of the protocols, it can be said that a possible key to oppose the emergence of the intention-behavior gap based on lack of availability is a better planning of the daily nutritional routine. The high number of codings also reflects the importance of this factor. It suggests that people should not rely on ready-to-eat offers when traveling, but rather prepare food at home and take it with them. Planning should also involve obtaining information about where to find sustainable food offers, whether in the supermarket or in restaurants. Therefore, information needs to be available as well. This becomes especially important when people are traveling to foreign cities. According to these results, both external and internal factors play a role in order to tackle the influencing factor availability. Availability is a strong factor overall that affects our food choices, because it is not possible to consume particular products that aren't offered (79–85). This supports the recommendations made elsewhere (12, 86–88) that we generally need to create a higher availability of sustainable food offerings. As a successful example we mention organic food purchasing in Denmark, where the availability as well as the assortments of organic food in typical supermarkets and discounters has been increased and where the promotion efforts of organic food products has been intensified (89). Improving the availability of sustainable and health promoting food can have a positive influence on people's intention-behavior relation and support the practice of a sustainable diet (90).

#### Education

Data for the second external factor education showed us that the most important thing about education seems to be gaining knowledge, as maintained by the participants. Thereby this external factor has a direct link to the internal factor knowledge. Personal knowledge is also the basis to develop skills for purchasing, cooking and preparing food. Participants stated that education about food system related topics should be implemented in school lessons, so that children can get in contact with the topics from an early stage. People also need to be proactive and engage with education offers. Gaining knowledge can increase awareness and build personal norms and values that are important for one to build a reliability during purchase actions. What we can read from the protocol data is also that education needs to involve providing information about food labeling, so that people are aware of the meaning of food labels. Food labeling itself should offer information about the product's background, such as production, origin, etc. and be a credible and reliable indication, so that people can easily notice, read and understand the labeling. To achieve this, the engagement of various actors including manufactures, retailers and public bodies is needed (78). Participants said that even supermarkets themselves can become part of the education system as providers of information by posting information about the products at the point of sale. Education or lack of it was assessed to have a strong influence on causing the intention-behavior gap. What is interesting is that over half of the participants say that education is not responsible for causing their personal intention-behavior gap, however, that is the case for just under half of the participants. The question therefore arises whether education is also equated with lack of information and transparency, such as having exact information about the individual products in the supermarket. According to the results from the questionnaire participants see the need for more educational work in schools, as well as through media and information distribution at the point of sale. People cannot make informed choices when they have insufficient knowledge (79), which is why education can have a significant influence on our consumption behavior (82). Therefore, it is necessary to implement further educational work on sustainable diets and food systems by media, food stores or restaurants, private organizations and governments for instance through awareness raising campaigns (91). Since previous educational work hasn't always been successful, a rethinking of new paths must also be made here (92). It seems promising when people act as multipliers. Influencers can also play an important role in digital times, especially with regard to the young population (93).

#### Advertising

The third external factor was advertising. During the think aloud sessions participants often said that advertising influences our subconscious and that is has a strong power over our behavioral control. Also, because people are confronted with it via multiple channels and there is a corresponding availability of the advertised products, it makes it very hard to resist. People who are hungry while they are out shopping for groceries are especially easy to influence. Participants said that in general it is very difficult to resist advertising strategies from companies. These statements are interesting in juxtaposition with the results of the supplementary questionnaires. Participants assessed advertising influence as rather moderate to fairly strong in causing the intention-behavior gap. This result stands out against the other three factors. Resisting the influence caused by advertising demands distinct consciousness and strong willpower to be able to do so. Another strategy can be using advertising strategy to promote sustainable food and sustainable diets. This approach could translate the “unwanted and negative” consequences of advertising into positive ones and strengthen the intention-behavior relation. Since people are usually exposed to a wide range of advertisements in magazines, on radio, TV and billboards, food that is not promoted that intensively, like fresh unpacked vegetables and fruits, possibly don't appear to be that attractive to buy. The strong influence of advertised fatty, salty, sugary snacks and drinks (87) is quite omnipresent and therefore it may be interesting to use similar advertising techniques for sustainable food products. Pollard et al. state that maybe more innovative advertising strategies are needed to promote health promoting and sustainable food (81).

#### Price

The price of food was the fourth external factor that was investigated. From the gathered data it becomes very clear that the price has a strong influence on causing the intention-behavior gap and has a direct link to the individual financial situation of people and their income. The influence may differ amongst people (81), but for those who just don't have enough money at hand, this can be a limiting factor. Of course, this can be for different reasons, but the social environment can also play a role here. For example, if a person has to provide for a whole family, the budget has to be shared among several people. What also plays an important role when dealing with higher food prices seems to be personal norms and values, as maintained by the participants. People need an understanding of values i.e., that food is worth spending money on. This understanding goes hand in hand with consciousness, which can also be seen by reference to the codings. Consciousness is needed, because participants also said that the thought of saving money is in some way part of people and to get rid of it seems to be very difficult. In order to eliminate price as an influencing factor, it seems helpful for individuals to manage the monthly budget that can be spent on food and for this reason also to rethink the diet composition. In this way people may save money that they can spend on higher quality food. It is also necessary to offer educational work that informs people why the price of, for instance, organic food is higher than for non-organic. Through this educational work, knowledge and thus consciousness can be built up and strengthen the intention-behavior relation by having control over the behavior. Higher prices for sustainable food like organic are still a main barrier (94, 95) and crucial for people's daily food choices (80, 96). Therefore, it seems to be important in order to support the practice of sustainable diets, that people don't have to struggle with higher food prices (96). It follows that one part of the solution here can be a realistic pricing of food forced by policies. The concept of internalizing external costs into food prices is not new and would indicate what food is produced in a more sustainable way and what food in a less sustainable way. Today a higher price is an indicator of a higher quality (97). This would then be the other way around.

### General Impact of Internal and External Factors

In addition, we gained further general insights into the impact of external and internal factors influencing behavioral control. Results from the follow-up interview show that the four external factors availability, education, advertising and price can have a big influence on behavior, at least according to the participants, because all twenty said that it would definitely be easier to consequently practice a sustainable diet, if the four factors weren't interfering as barriers. Some of the participants have thereby focused on individual factors and allocated them a particularly strong influence. Not all participants were able to list other external factors, when we asked for them. Twelve participants identified the social environment as an influential factor. Other factors were food packaging (three mentions), time (two mentions), attractivity (one mention) and labeling (one mention). It remains unclear if the participants spontaneously had no idea, whether they were simply unaware of other external factors, or if other external factors don't exert the similar strong influence on behavioral control, such as the four chosen factors that were explored. To find out more about the importance of external factors as influences on behavioral control we then asked participants if they agreed with the saying that others have to change something first before they can change something, which alludes to external factors as a key condition. What is interesting is that answering this question didn't seem to be easy, because some participants didn't give a clear answer and could not decide definitively on one answer, but rather saw it on the one hand and on the other. From the answers to the first question we get the impression that external factors have a strong influence on people's behavioral control and thereby create the intention-behavior gap. Relating this impression to the answers of the third question, then the answers of the participants would be in some way contradictory, because here the participants still see the responsibility with them. On the other hand, it could be said that the participants see the responsibility as their own, but the control of their own behavior cannot withstand the strong influence of external factors. Therefore, the results of the last interview question may help this debate further. While ten participants stated that both external and internal factors are decisive in causing the intention-behavior gap, four said definitely that external factors are more decisive and six participants are convinced that internal factors are more decisive. This shows a small majority attributing the greater influence to the internal factors. Nevertheless, the external factors are also said to have a significant influence on the behavior and therewith on the intention-behavior gap, if they are, for example, a limiting necessity, like in terms of availability.

### Interaction With Internal Factors

Internal factors play an important role within every examined external factor. The factor personal norms and values was present with a relatively high coding within all four factors' protocol data. This can indicate that people need to live up to their personal norms and values so that external factors cannot evolve such a strong influence on their behavior control. Looking at Ajzen's model of the TPB (Figure 1) we find values within the background factors. Moral and personal norms have a proven impact on people, so that they are acting according their intentions (98, 99). This can be illustrated by one participant's quote in this study. The participant said that quality always comes first for him. By forming this strong personal norm or value he can eliminate the higher food price as a possible barrier. Therefore, it can be key in suppressing the emergence of the intention-behavior gap to establish personal norms and values that are in line with what sustainable food systems stand for.

Planning as an internal factor shows the highest number of codings within the protocol data on availability. According to literature the intention-behavior gap can in many cases be bridged through planning (100). The intention-behavior relation can be generally strengthen by planning and implementation intentions (101) which are, essentially, verbalized if-then plans that are formed to enhance the translation of intentions into behavioral action (102).

What is also striking is that knowledge is the most frequent coding within the education protocol data. Gaining knowledge seems to be considered essential for making informed decisions, but people don't always apply their knowledge when acting (90). It may become clearer that one factor alone cannot have such a strong positive influence, and stands always in interaction with others. Although this still stands to debate. Knowledge can also be found as a background factor within the model of the TPB.

Willpower is, besides hunger, the most frequently coded factor within the protocol data about advertising. People's lack of willpower is often mentioned when try to stay on a diet or withstand external influences (103). To be able to resist external influences, for instance unhealthy food offerings or the social environment, requires willpower and also strong conviction of the planned behavior.

Within the price protocol data finances are the most frequent coding. In society the personal finance situation can limit access to many things in life, especially food. For most people, the financial situation mainly depends on the income, which is also listed as a background factor within the TPB model.

**Figure 3:**
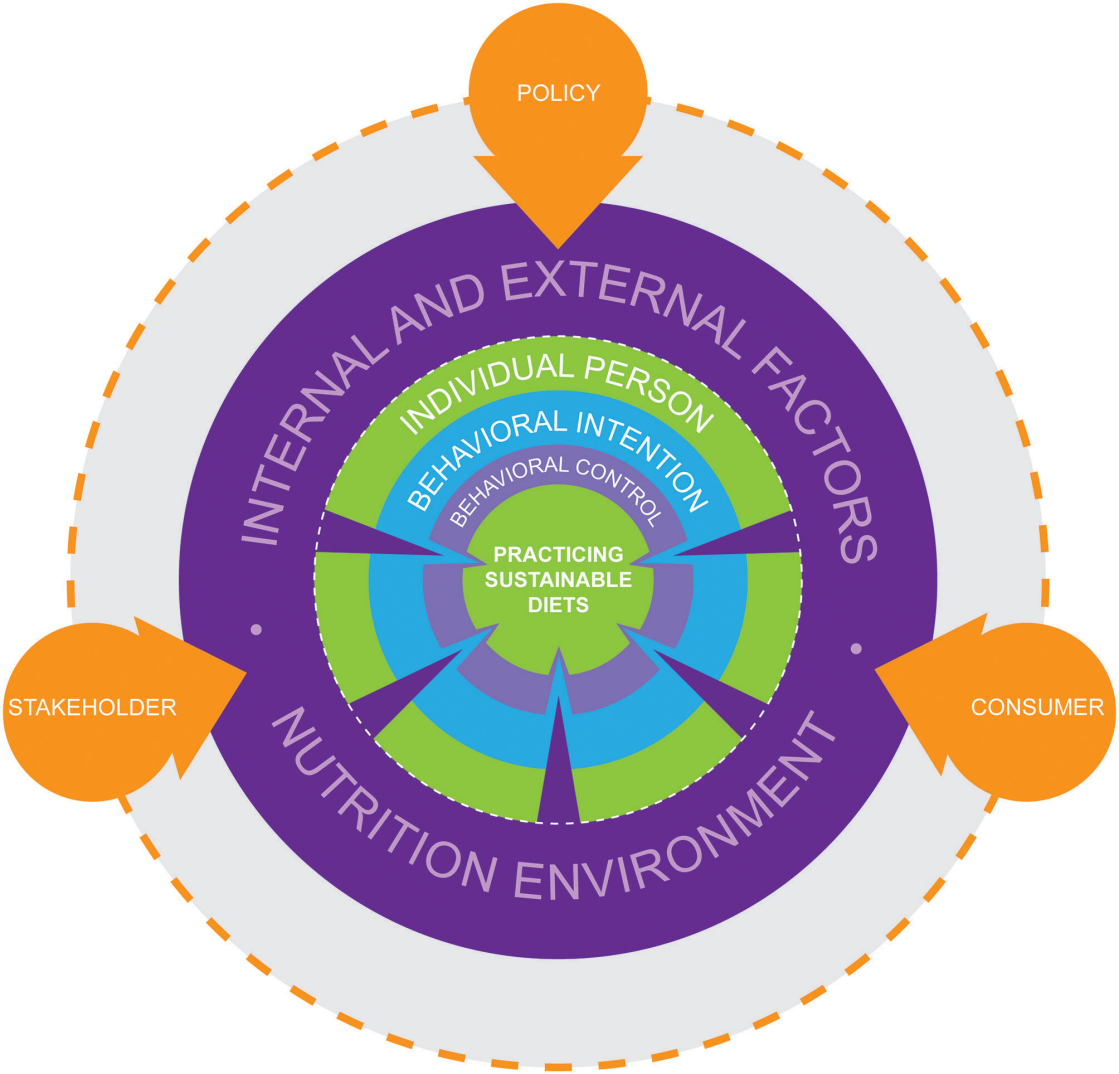
Actors supporting people practicing sustainable diets.

What the results and this discussion show is that all four external factors interact in some way with one another and also with other external and internal factors (Figure 2). This in turn opens up different approaches to dealing with the influencing factors. Therefore, approaches to solutions cannot always be one-dimensional, as would be, for example, the approaches there must be a higher availability of sustainable food products, there must be more education for sustainable diets and food systems, there must be less advertising for unsustainable food products, and there must be lower prices for sustainable food products. The factors can be tackled with different interacting strategies from different actors. Policy, stakeholder and consumers themselves can shape external as well as internal factors (which form the nutrition environment) that positively influence people's behavioral intention and behavioral control and thereby support the practice of sustainable diets (Figure 3).

Finally, we can say that by applying a mixed methods approach we were able to gain valuable data on our four chosen external factors. Nonetheless, a few limitations have to be listed for this study. First, selecting our study sample was only based on people's willingness to participate. Besides, the study sample does not represent the general public. When asking if participants are trying to practice a sustainable diet, three participants negated it. It may be questioned whether people who are not trying to practice a sustainable diet are able to make more valuable contributions about factors that can interfere with the intention to do so. Furthermore, we used video-simulations that participants watched during the think aloud sessions. The fictional stories used to represent the external factors in the videos may have had too much impact on the participants' verbalized thoughts, as we don't know whether they have made the same experiences as shown in the videos. Therefore, it may have been better to accompany the participants in their daily life (e.g., during groceries shopping, traveling or family dinners). However, this turns out to be rather difficult in the execution. In addition, we refrained from sitting in on the sessions and constant reminding of the participants to think aloud. Upon request, the participants preferred to be alone in the room because they felt more comfortable this way. We also haven't used a control group, because we didn't aim for a comparison. Nevertheless, it should be noted that the recruitment of participants from the nutritional section at the University of Applied Sciences could have an impact on the results.

## Conclusion

In order to establish sustainable food systems and especially eliminate ongoing environmental threats, we need a higher adoption rate of sustainable diets. Because in our existing food systems we are surrounded by factors that influence our behavioral control it is important to offer people support for enhancing the practice of sustainable diets. Therefore, the aim of this study was to explore the external factors availability, education, advertising and price that can cause the emergence of an intention-behavior gap while people are trying to nourish themselves sustainably. The objective was to gain a better understanding of the factors in order to be able to make more targeted and valuable recommendations for policy, stakeholder and consumer action.

**Figure 4:**
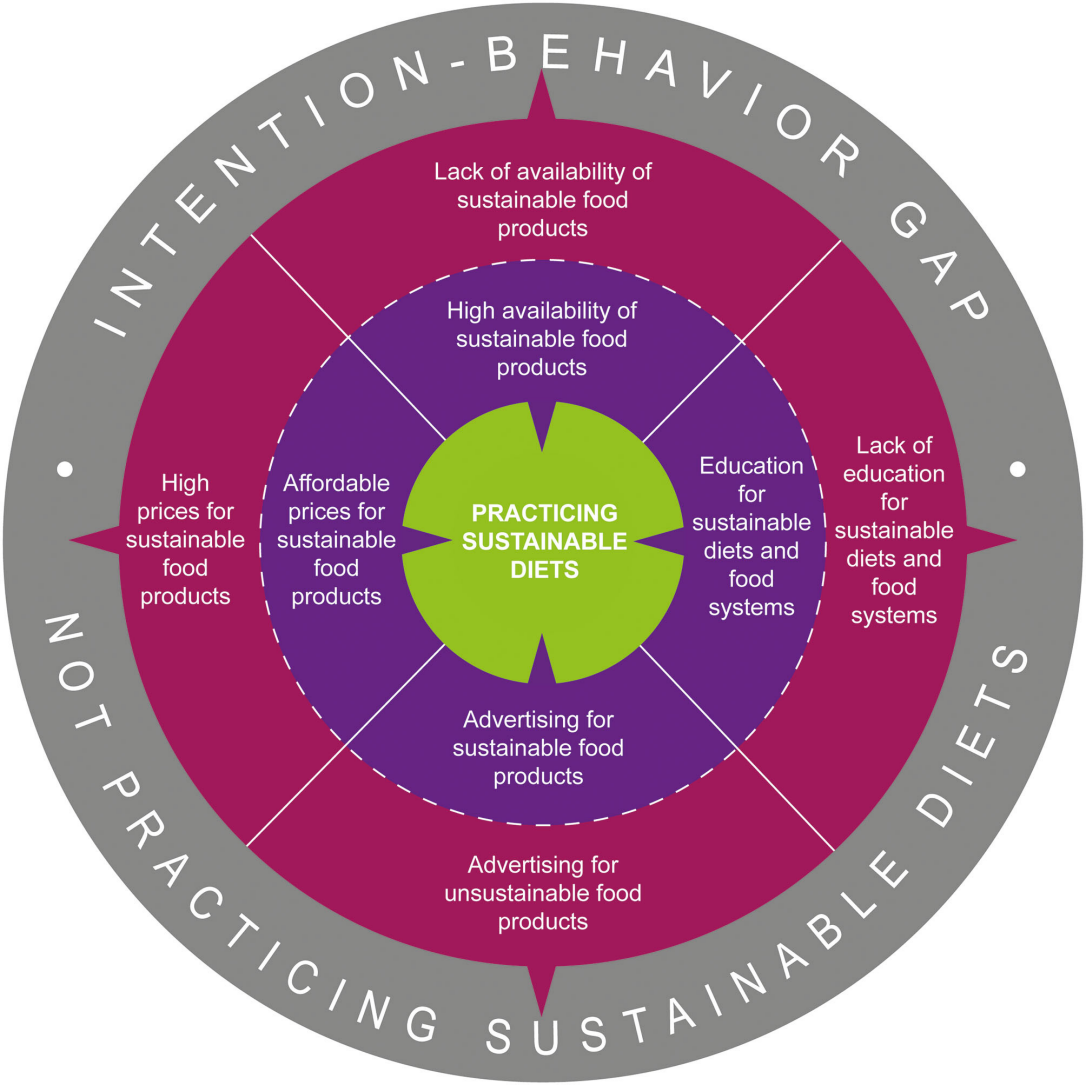
Practice of sustainable diets influenced by the shape of the factors availability, education, advertising and price.

Within this research we were able to analyze and understand the factors availability, education, advertising and price more closely. The findings of our study show that depending on the shape of the factors, they can cause the intention-behavior gap and lead people to not practicing sustainable diets or they can support peoples' practice of sustainable diets (Figure 4).

Hence, external factors can have a decisive influence on our behavioral control and also represent limiting factors that stand in the way of performing a sustainable diet. That is the case for the external factor availability. If sustainable food isn't available to people, they cannot buy and eat it. Therefore, a corresponding availability should be given in order to meet the possible and future demand. Policy action should on the one hand target subsidies for sustainable food production and establish sustainability standards to build a basis for a higher availability of sustainable food products (104). On the other hand, governmental action should address public food procurement. Hence, this call for more availability is also aimed at all producers, distributors, persons in charge in the food retailing industry and in out-of-home foodservice such as restaurant chefs or catering directors to produce and offer more sustainable food products. Since restricting peoples' choices is not always a welcomed way to go, nudges can be implemented – especially for reducing meat consumption in public procurement (87, 105). Nudges are known for their potential of promoting healthy and sustainable diets within public procurement. They can be implemented for example in the form of acoustics, lighting, reward, commitment, role models, or dishes, food presentation or trigger food (106). For targeting time pressure of people as a barrier for practicing sustainable diets, special services can come with sustainable food offers, like express service during lunch breaks (107). Nudges for supermarkets can target visual cues like the size of a display area and the amount of sustainable food products displayed. This can lead to a higher purchase of sustainable food through more visibility (108). Providing retailers, supermarkets, food stores etc. with financial incentives that come with offering sustainable food products can be attractive to increase the availability, as people will come and buy (104). Here, communication and information distribution become important. People need to know where to find sustainable food products. Therefore, providing them with information in the form of a list or a mobile app of sustainable food offers and where to find them, can be helpful. The resulting geographic overview of supermarkets, stores, farmers markets, etc. also makes it clear that there must be a good distribution of shopping facilities to improve access to sustainable food for all people (109). What is also worth mentioning is that not only the amount of sustainable food products should be increased but also the variety in terms of biodiversity (109). As long as there is no ubiquitous availability of sustainable food products, people have to plan their daily nutrition and inform themselves about possible sources of purchase (whether in the supermarket or in out-of-home foodservice) in advance or do meal prepping at home for lunch breaks, travels etc. Depending on the possibilities, a garden for vegetables, fruits or herbs can be laid out. Therefore, gaining knowledge through education seems essential. But also support by the media is needed, especially for informing about food offers via advertising or mobile apps.

The results further suggest that we need more educational work in general, that focus on food system education in schools but also on appropriate offers for adults. Without knowledge, it is difficult to make better buying decisions for sustainable food products in our current existing food systems.

Politicians, especially in education ministries, and official educational institutions are as much implicated in addressing this as are all employees and volunteers in educational settings and private individuals in their social environments. Education is essential for promoting awareness. Therefore, starting education in kindergarten and schools with topics like nutrition, cooking, gardening or school meals preparation can support awareness building (110). Education also includes providing information about food products and their backgrounds. Here, also retailers and supermarkets can support this by providing people with information at the point of sale to enable reasoned buying decisions. Restaurants and other out-of-home foodservices can inform their customers about the offered food and meals to contribute to people's education and consciousness development. Media play, as mentioned before, an important role to inform people where to find sustainable food products, inform about food labeling or strategies for practicing sustainable diets in everyday life. Here, influencers can play a key role—especially for younger generations—by talking and posting about sustainable lifestyles and practicing sustainable diets. At the same time, people need to actively seek out and take advantage of existing educational opportunities. Otherwise, practicing sustainable diets without needed skills for shopping and cooking, knowledge or consciousness will become difficult.

Food advertising is mainly used for fatty, salty, sugary snacks and drinks (111–113). Different advertising strategies successfully influence people's nutrition behavior. It could be promising to use these for sustainable food to influence people like it is already done for other public interests like stop smoking, use condoms or NGO work related ads like from Greenpeace. In order to initiate a corresponding change, political action is needed, especially with regard to advertising for children's food (114–117). Only restrictions or bans can reduce people's (and especially children's) exposure to the advertising of unhealthy and unsustainable food products (118, 119). Advertised food products are mainly brand-name products that have a high availability in almost every store. Therefore, stakeholders like NGO's, producers (using farmer-to-consumer direct marketing) and companies from the sustainable food industry have to think about showing initiative and consider to use similar offensive advertising strategies and at the same time offering a higher availability of sustainable food products. Above all, strategies have to be developed for fresh foods, such as fruits and vegetables, which usually do not belong to a well-known brand or are advertised at all, so that these foods do not remain anonymous, but instead become more attractive to buy. By promoting these foods and sustainable food products in general, advertising and communication strategies should target values, so that people can relate to the products (109). Media as well as influencers and celebrities can be used to promote sustainable food products and make a positive contribution to enhance the practice of sustainable diets. Especially to reach today's youth who live in a media-saturated environment, multiple channels and techniques need to be used as marketing efforts that serve their health (120). Meanwhile consumers need education to be able to identify advertising for unsustainable food products and not to be negatively influenced by strong advertising efforts. Therefore, a strong willpower is needed that can be supported by the social environment of friends and family. The extension of the social environment to the overall nutrition and food environment paints the picture of the structures in which we live and where it can be difficult to practice a sustainable diet. Individual practice of sustainable diets can only occur in a supportive environment, where sustainable food products are accessible and affordable (120). Therefore, the settings like school, home, work sites, retail food stores, convenience and corner stores, supermarkets, fast food outlets, restaurants, neighborhoods and communities are as important as are family, friends and peers, that build our nutrition environment (120, 121). What is striking is that these influential factors, especially food prices, availability and offers in our neighborhoods can have a direct link to the health status of people living in it (120).

The higher prices of sustainable food are a long-burning issue. It may seem that some people cannot overcome the price barrier or in other cases they simply cannot afford to spend more money on sustainable foods. But buying cheap food and practicing poor diets comes with higher follow-up cost for healthcare and repairing environmental damage (if at all possible) (122). According to research UK consumers have actually to pay twice as much for their food when considering the true costs for diet-related diseases and natural capital (122). A true cost accounting for food can transform food systems when food products reflect their true costs (123). This policy action would solve the awareness deficiencies of people and would probably immediately increase the willingness to buy sustainable food products. Just reducing food prices for sustainable products without using policy subsidies would be disastrous for producers. Another approach is to subsidize healthy and sustainable food products and to tax unhealthy and unsustainable food products (124–130). The latter has already been done in some countries, like for example the tax on sugary drinks in Mexico or a fat tax in Denmark (131–136). What can be said is that sustainable food should be affordable for all people. Otherwise, a large part of society cannot be expected to buy them. Among others, maybe politicians, producers, traders and economic experts can manage to agree on price models for food (e.g., cost sharing arrangement based on income) that can be implemented in the future. Meanwhile, people, within their financial resources, can try to show willingness to pay the supposedly higher prices for sustainable food products. Here again the link to education and resulting knowledge about food and their costs can build consciousness which leads to the willingness to pay. Also targeted advertising strategies can have a positive impact on the willingness of people to pay more for sustainable food products (137).

What arises from our findings is that we need innovations for system improvements and different interacting strategies to promote a behavior change toward health promoting and sustainable diets (37). Therefore, every stakeholder (farmers, industry, caterer, media, NGO's etc.) acting within food systems has a part to play and can think about making an effort and contribute to an improvement of our current food systems (36, 78). Therefore, we definitely need appropriate and supportive policy action in some cases that enable changes in the current system and structures (21) but also individual consumer efforts. We strongly encourage scientists to further investigate other external and internal factors that influence people's behavioral control to deliver applicable supportive strategies that bring about the necessary changes. It still is a major challenge to support people's behavior toward sustainable diets and establish sustainable food systems.

## Data Availability Statement

The datasets generated for this study are available on request to the corresponding author.

## Ethics Statement

Ethical review and approval was not required for the study on human participants in accordance with the local legislation and institutional requirements. The patients/participants provided their written informed consent to participate in this study.

## Author Contributions

AP had the idea for this study. LF and AP developed the idea further. LF, CS, and AP designed the study, analyzed the data, and wrote the manuscript. LF created the videos, conducted the study, transcribed the think aloud protocols, and follow-up interviews. CS did the final language editing. All authors contributed to the article and approved the submitted version.

## Conflict of Interest

The authors declare that the research was conducted in the absence of any commercial or financial relationships that could be construed as a potential conflict of interest.
